# Gallbladder and biliary tract cancer burdens in China from 1990 to 2021 and projection to 2044: findings from the 2021 Global Burden of Disease study

**DOI:** 10.3389/fmed.2025.1592621

**Published:** 2025-05-09

**Authors:** Zhongyi Jiang, Qianwei Jiang, Pusen Wang, Lin Zhong

**Affiliations:** ^1^Department of General Surgery, Shanghai General Hospital, Shanghai Jiao Tong University School of Medicine, Shanghai, China; ^2^Department of Liver Surgery and Organ Transplantation Center, Shenzhen Third People’s Hospital, Second Affiliated Hospital, Southern University of Science and Technology, Shenzhen, Guangdong, China; ^3^Department of Gastric Surgery, Zhejiang Cancer Hospital, Hangzhou, Zhejiang, China

**Keywords:** gallbladder and biliary tract cancer, disease burden, epidemiology, ASR, EAPC

## Abstract

**Background:**

Gallbladder and biliary tract cancers (GBTCs) have high incidence and mortality rates in China, imposing a substantial disease burden. Establishing comprehensive strategies is crucial for alleviating this burden. We report the average estimated annual percentage change (EAPC) in age-standardized rates (ASRs) of GBTCs globally and in China via Global Burden of Disease (GBD) study 2021 data and the relationships of GBTCs with body mass index (BMI) and the sociodemographic index (SDI). The 2021–2044 GBTCs prevalence trends were predicted by sex.

**Methods:**

We collected and analyzed GBD data from 1990 to 2021, including incidence, prevalence, deaths, disability-adjusted life years (DALYs), and age-standardized incidence (ASIR), prevalence (ASPR), mortality (ASMR), and DALYs (ASDR) rates and calculated the proportion of deaths and DALYs attributable to risk factors by sex. Future trends from 2022 to 2044 were predicted with Nordpred age–period–cohort models.

**Results:**

The number of GBTCs-related incident cases, prevalent cases, deaths, and DALYs increased from 1990 to 2021. However, the GBTCs-related ASMR and ASDR decreased during the same period. These changes may be related to risk factors, such as increased BMI. Furthermore, we used the projection model to estimate that the GBTCs-related ASPR in all populations will steadily increase, whereas the GBTCs-related ASMR and ASDR are expected to significantly decline until 2044.

**Conclusion:**

The increasing GBTCs prevalence in China due to SDI advancements and population aging underscores the importance of early monitoring and prevention measures to alleviate the disease burden.

## Introduction

Gallbladder and biliary tract cancers (GBTCs) are a heterogeneous and aggressive group of adenocarcinomas characterized by high morbidity and mortality rates (1, 2). Only a subset of patients with early-stage GBTCs can achieve a cure through surgical resection or liver transplantation (under stringent selection criteria); however, the majority of GBTC patients present with incurable locally advanced or metastatic disease due to delayed detection and thus have an exceedingly unfavorable prognosis, with only 2–5% for 5-year overall survival (3–5). In 2020, there were 115,949 new cases and 84,695 new deaths from GBTCs worldwide, ranking 25th and 21st among the 36 cancers, respectively (6). Despite accounting for only approximately 3% of all digestive system tumors, GBTC still imposes a significant societal and economic burden due to its unfavorable prognosis (7).

The burden of disease evaluation currently serves as a comprehensive assessment system for quantifying the health impact of diseases and risk factors across diverse geographical regions, generations, age groups, and sexes. The distribution of GBTCs varies significantly globally, and the mortality rates of GBTCs have exhibited a downward trend in most countries worldwide. Certain high-income nations have experienced an upward trajectory in recent years (8–10). Moreover, previous studies have shown that the incidence and mortality rates of GBTCs are typically higher in Asia and South America than in high-income countries, such as China and Thailand, where the incidence is 40 times higher (1, 11, 12). China poses a significant risk for GBTCs. According to the Global Burden of Diseases (GBD) 2019 study, China contributes to approximately one-fifth of the global disease burden in terms of new cases and deaths. Additionally, from 1990 to 2019, there has been a consistent upward trend in the age-standardized rates of incidence, prevalence, mortality, and DALYs for GBTCs in China (10).

This study utilized comparable data extracted from the latest database of GBD 2021 Study to analyze the prevailing disease burden trends in GBTCs over three decades in China and project future disease burdens for the next two decades (13). The findings will assist healthcare professionals and health organizations in comprehending the epidemiological shifts of GBTCs from a public health perspective, as well as the impact of changing lifestyles and preventive policies on recent trends and the current burden of GBTCs. These insights can further provide comprehensive analyses and forecasts for developing public health policies, prevention strategies, and treatment interventions.

## Methods

### Data sources

The GBD 2021 is a publicly accessible database coordinated by the Institute for Health Metrics and Evaluation (IHME) that includes comprehensive data on mortality and disability in 204 countries and territories, including 288 causes of death, 371 diseases and injuries, and 88 risk factors worldwide. In this study, we extracted data on incidence, prevalence, mortality, disability-adjusted life-years (DALYs), and risk factors for GBTCs in China and globally across sex and age groups from 1990 to 2021 using the Results Tool of the GBD 2021.^1^ The projected demographic data from a forecasting analysis for the GBD study by Vollset et al. (14) were further used to forecast the age-standardized rates (ASRs) of GBTCs in both sexes from 2022 to 2044. The Socio-demographic Index (SDI) is a composite indicator of a country’s lag-distributed income per capita, average years of schooling, and the fertility rate in females under the age of 25 years. The classification criteria for SDI are as follows: low SDI: 0.0000–0.4658; low-middle SDI: 0. 4,658–0.6188; middle SDI: 0.6188–0.7120; high-middle SDI: 0.7120–0.8103; high SDI: 0.8103–1.0000. SDI values range from 0 to 1, with higher values indicating higher socioeconomic development (15, 16).

In the present study, we used data from the GBD Study 2021, which was approved by the institutional review board of the University of Washington School of Medicine. As this is a secondary analysis of existing data, no additional human research ethics review or informed consent was needed. The original data collection obtained informed consent from the study participants or was granted exemptions by the institutional review board. Study data were anonymized and deidentified to protect the privacy and confidentiality of the study participants.

### Statistical analysis

The estimated average percentage change (EAPC) was calculated to assess the trend of ASR for the burden of GBTCs, where the ASR trended upward if the EAPC value and the lower limit of the 95% confidence interval (CI) were greater than 0, downward if these values were less than 0, and constant when these values equaled 0. On the basis of the power5 age–period–cohort model, Nordpred age–period–cohort (NAPC) analysis was used to project the ASRs of incidence, prevalence, mortality, and DALYs for GBTCs by sex from 2021 to 2044 in China. The data analyses were conducted via R (version 4.4.1) and GraphPad Prism 9 software, whereas the “Nordpred (version 1.1)” package was used for the NAPC predictive model. *p* < 0.05 was considered to indicate statistical significance.

## Results

### Incidence and prevalence burdens of GBTCs in 2021

**Figure 1:**
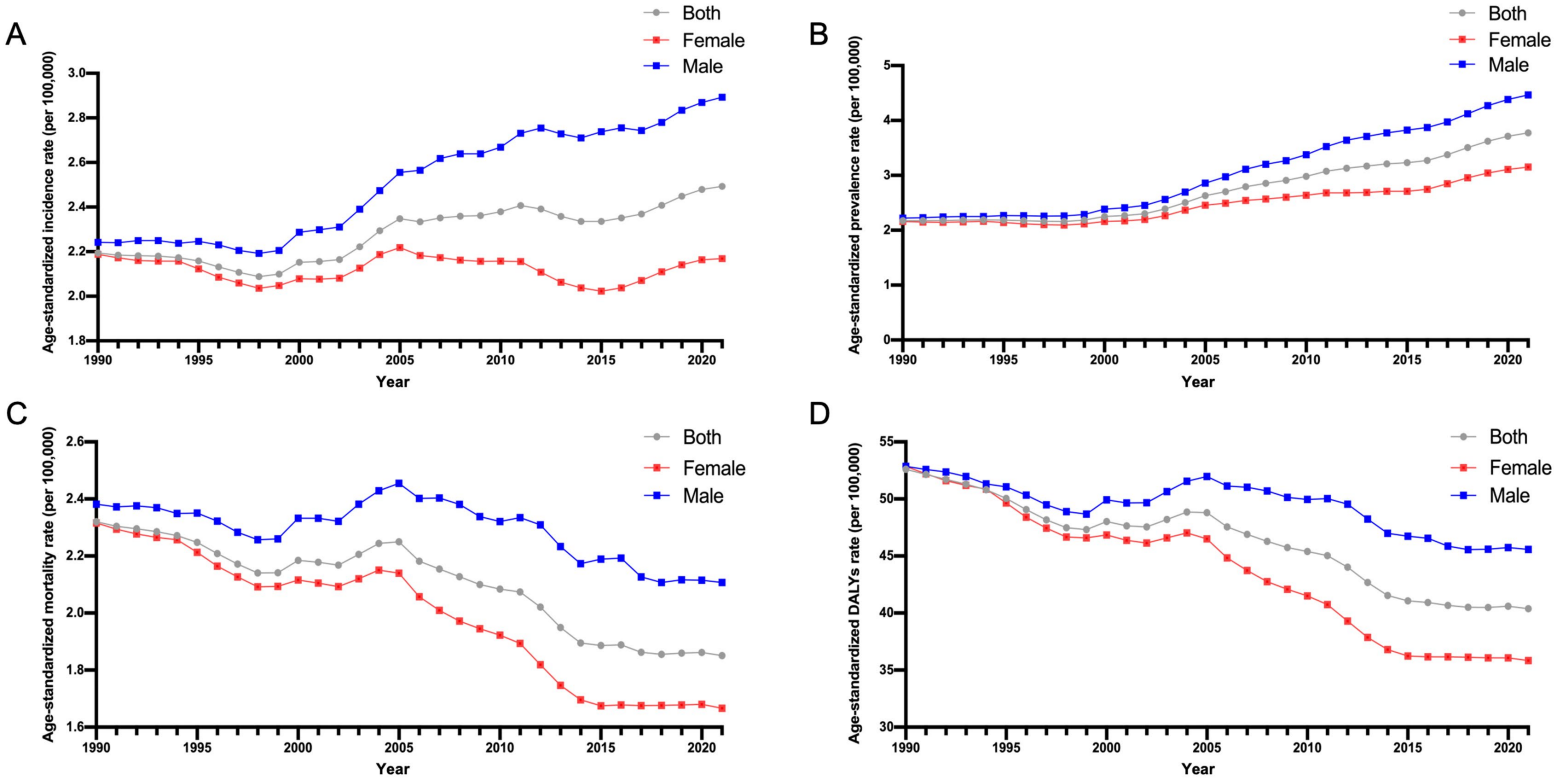
Age-standardized incidence, prevalence, mortality, and DALYs rates of GBTCs in China from 1990 to 2021. **(A)** The ASIR of GBTCs. **(B)** The ASPR of GBTCs. **(C)** The ASMR of GBTCs. **(D)** The ASDR of GBTCs. ASIR, age-standardized incidence rate; ASPR, age-standardized prevalence rate; ASMR, age-standardized mortality rate; DALYs, disability-adjusted life years; ASDR, age-standardized DALYs rate; GBTCs, gallbladder and biliary tract cancers.

**Figure 2:**
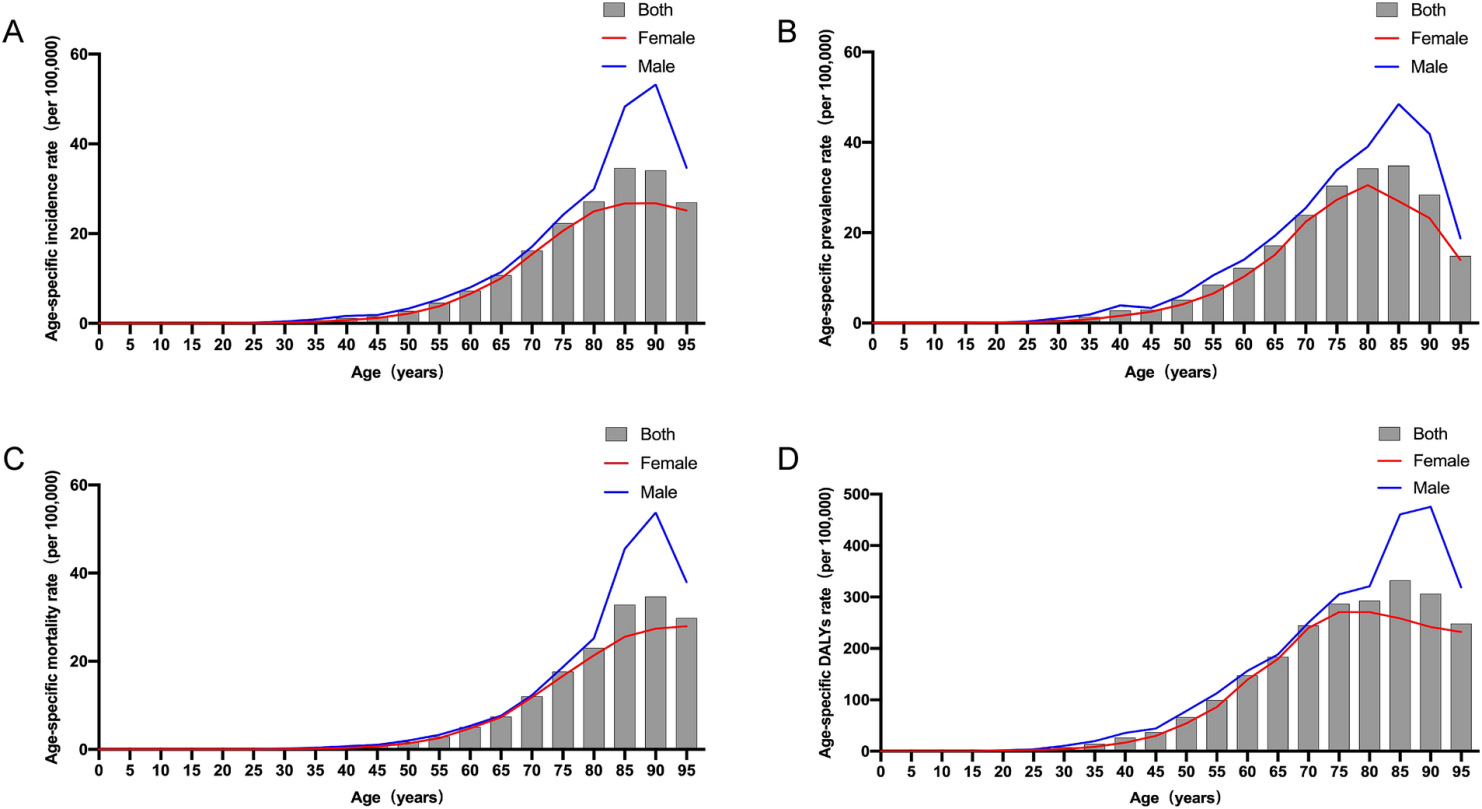
Age-specific incidence, prevalence, mortality, and DALYs rates of GBTCs by age and sex in China in 2021. **(A)** Age-specific incidence rate of GBTCs. **(B)** Age-specific prevalence rate of GBTCs. **(C)** Age-specific mortality rate of GBTCs. **(D)** Age-specific DALY rate of GBTCs. DALYs, disability-adjusted life years.

The incidence of GBTCs has significantly increased from 17.08 (×1,000) in 1990 to 51.72 (×1,000) in 2021 in China, with the ASIR increasing from 2.19 per 100,000 populations in 1990 to 2.49 per 100,000 populations in 2021 (Table 1). The increase was more pronounced among males than females, with incident cases showing a remarkable 3.42-fold increase in males (EAPC = 1.00; 95% CI = 0.88 to 1.11) and a 2.68-fold increase in females (EAPC = −0.04; 95% CI = −0.14 to 0.06) (Table 1; Figure 1A). The highest age-specific incidence rates (per 100,000 populations) for both males and females were consistently observed within the 90–94-year-old age group (Figure 2A). Similarly, there has been a remarkable increase in the prevalence of GBTCs, with a significant increase from 17.98 (×1,000) in 1990 to 79.69 (×1,000) in 2021, accompanied by an increase in the age-standardized prevalence rate (ASPR) from 2.18 per 100,000 populations in 1990 to 3.77 per 100,000 populations in 2021 (Table 1). The number of prevalent cases exhibited a significantly increased 5.10-fold among males (EAPC = 2.62; 95% CI = 2.42 to 2.83) and 3.80-fold among females (EAPC = 1.35; 95% CI = 1.21 to 1.50) (Table 1; Figure 1B). In addition, the age-specific prevalence rate (per 100,000 populations) among males peaked in the 85–89-year-old age group, whereas among females, it peaked in the 80–84-year-old age group (Figure 2B).

**Table 1:** Incidence and prevalence of GBTCs in 1990 and 2021 and their temporal trends from 1990 to 2021.

		Prevalence					Incidence				
		Numbers ×1,000 (95% UI)		ASR per 100,000 (95% UI)		EAPC (95% CI)	Numbers ×1,000 (95% UI)		ASR per 100,000 (95% UI)		EAPC (95% CI)
	Sex	1990	2021	1990	2021	1990–2021	1990	2021	1990	2021	1990–2021
China	Both	17.98 (13.59–22.71)	79.69 (53.15–103.93)	2.18 (1.66–2.74)	3.77 (2.51–4.91)	2.04 (1.86–2.22)	17.08 (13.00–21.74)	51.72 (35.62–66.85)	2.19 (1.68–2.79)	2.49 (1.71–3.21)	0.50 (0.40–0.60)
	Female	9.19 (6.28–12.80)	34.89 (21.15–47.65)	2.16 (1.48–3.00)	3.15 (1.91–4.30)	1.35 (1.21–1.50)	8.94 (6.15–11.48)	23.92 (14.70–32.56)	2.18 (1.51–3.05)	2.16 (1.33–2.95)	−0.04 (−0.14–0.06)
	Male	8.79 (5.77–12.29)	44.80 (25.71–62.88)	2.22 (1.45–3.16)	4.47 (2.58–6.19)	2.62 (2.42–2.83)	8.14 (5.38–11.48)	27.80 (16.11–38.97)	2.24 (1.46–3.23)	2.89 (1.69–3.40)	1.00 (0.88–1.11)

ASR, the age-standardized rate; EAPC, estimated average percentage change, CI, confidence interval; UI, uncertainty interval.

### Deaths and DALYs burdens of GBTCs in 2021

The number of deaths and DALYs attributed to GBTCs exhibited an increasing trend in 2021, with 37.83 (× 1,000) deaths and 857.50 (× 1,000) DALYs recorded, respectively (Table 2). In contrast, the ASMR and age-standardized DALYs rates in China declined from 1990 to 2021, with EAPCs of −0.65 (95% CI = −0.72 to −0.58) and −0.87 (95% CI = −0.96 to −0.78), respectively (Table 2). Notably, during this period, the decline trajectories of the ASMR and the age-standardized DALYs rate were more pronounced among the female population than among the male population (Table 2; Figures 1C,D). The highest age-specific mortality rate (per 100,000 populations) and DALYs rate for males consistently occurred in the 90–94-year-old age group, as depicted in Figures 2C,D. Conversely, among females, the age-specific mortality rate (per 100,000 populations) reached its peak within the >95-year-old age group, whereas the age-specific DALYs rate (per 100,000 populations) peaked within the 75–79-year-old age group.

**Table 2:** Deaths and DALYs associated with GBTCs in 1990 and 2021 and their temporal trends from 1990 to 2021.

		Death					DALYs				
		Numbers ×1,000 (95% UI)		ASR per 100,000 (95% UI)		EAPC (95% CI)	Numbers ×1,000 (95% UI)		ASR per 100,000 (95% UI)		EAPC (95% CI)
	Sex	1990	2021	1990	2021	1990–2021	1990	2021	1990	2021	1990–2021
China	Both	17.25 (13.21–22.14)	37.83 (26.65–49.26)	2.32 (1.78–2.97)	1.85 (1.29–2.40)	−0.65 (−0.72 – −0.58)	452.35 (341.84–581.40)	857.50 (601.93–1121.11)	52.61 (39.99–67.15)	40.38 (28.21–52.61)	−0.87 (−0.96 – −0.78)
	Female	9.17 (6.35–12.94)	18.31 (11.42–24.76)	2.32 (1.60–3.24)	1.67 (1.04–2.25)	−1.03 (−1.11–0.95)	230.96 (158.40–322.84)	397.71 (248.64–540.09)	52.85 (36.17–73.76)	35.83 (22.37–48.65)	−1.35 (−1.47 – −1.25)
	Male	8.08 (5.35–11.61)	19.52 (11.62–27.42)	2.38 (1.55–3.50)	2.11 (1.26–2.92)	−0.25 (−0.32 – −0.17)	221.39 (145.10–311.19)	459.79 (269.77–651.24)	52.86 (34.73–75.74)	45.57 (26.80–63.82)	−0.41 (−0.51 – −0.31)

ASR, the age-standardized rate; EAPC, estimated average percentage change, CI, confidence interval; DALYs, disability-adjusted life years; UI, uncertainty interval.

### Risk factors for GBTCs

**Figure 3:**
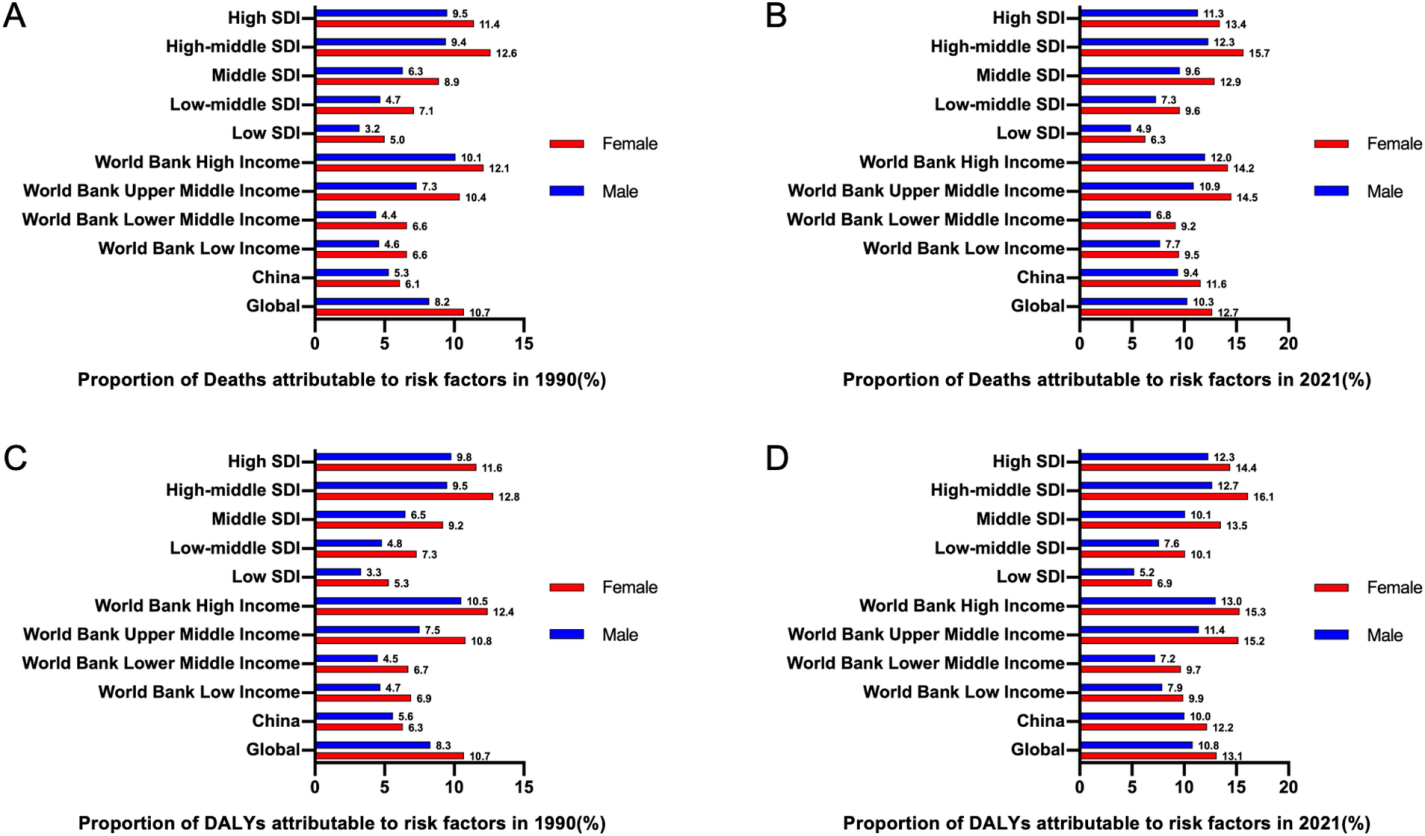
The proportions of deaths and DALYs attributable to risk factors associated with GBTCs globally, in China, and in 9 regions in 1990 and 2021 by sex. **(A)** The proportion of deaths attributable to risk factors associated with the development of GBTCs in 1990. **(B)** The proportion of deaths attributable to risk factors for the development of GBTCs in 2021. **(C)** The proportion of DALYs attributable to risk factors for the development of GBTCs in 1990. **(D)** The proportion of DALYs attributable to risk factors for the development of GBTCs in 2021. DALYs, disability-adjusted life years; GBTCs, gallbladder and biliary tract cancers.

Previous GBD studies have consistently demonstrated that an elevated body mass index (BMI) (>23.0 kg/m^2^) is a significant and influential risk factor for both deaths and DALYs in GBTCs worldwide. In this study, we conducted a comprehensive analysis to compare the proportion of deaths and DALYs attributed to high BMI risk factors from 1990 to 2021, specifically within China. In 1990, the proportion of deaths attributed to the high-BMI risk factor was merely 5.3% for males and 6.1% for females in China, which higher than the proportions observed in low SDI and World Bank Low Income regions, but fell significantly below the global average (Figure 3A). However, in 2021, the proportions increased to 9.4% for males and 11.6% for females, representing 80 and 100% increases, respectively, and these data lower than those observed in regions characterized by high-middle SDI and World Bank Upper Middle Income regions as well as the global average (Figure 3B). A similar trend can be observed in the proportion of DALYs attributable to high BMI risk factors. For males, this proportion increased from 5.6% in 1990 to 10.0% in 2021, whereas for females, it rose from 6.3 to 12.2%, which also lower than the global average (Figures 3C,D).

### Predictions of GBTCs from 2022 to 2044

**Figure 4:**
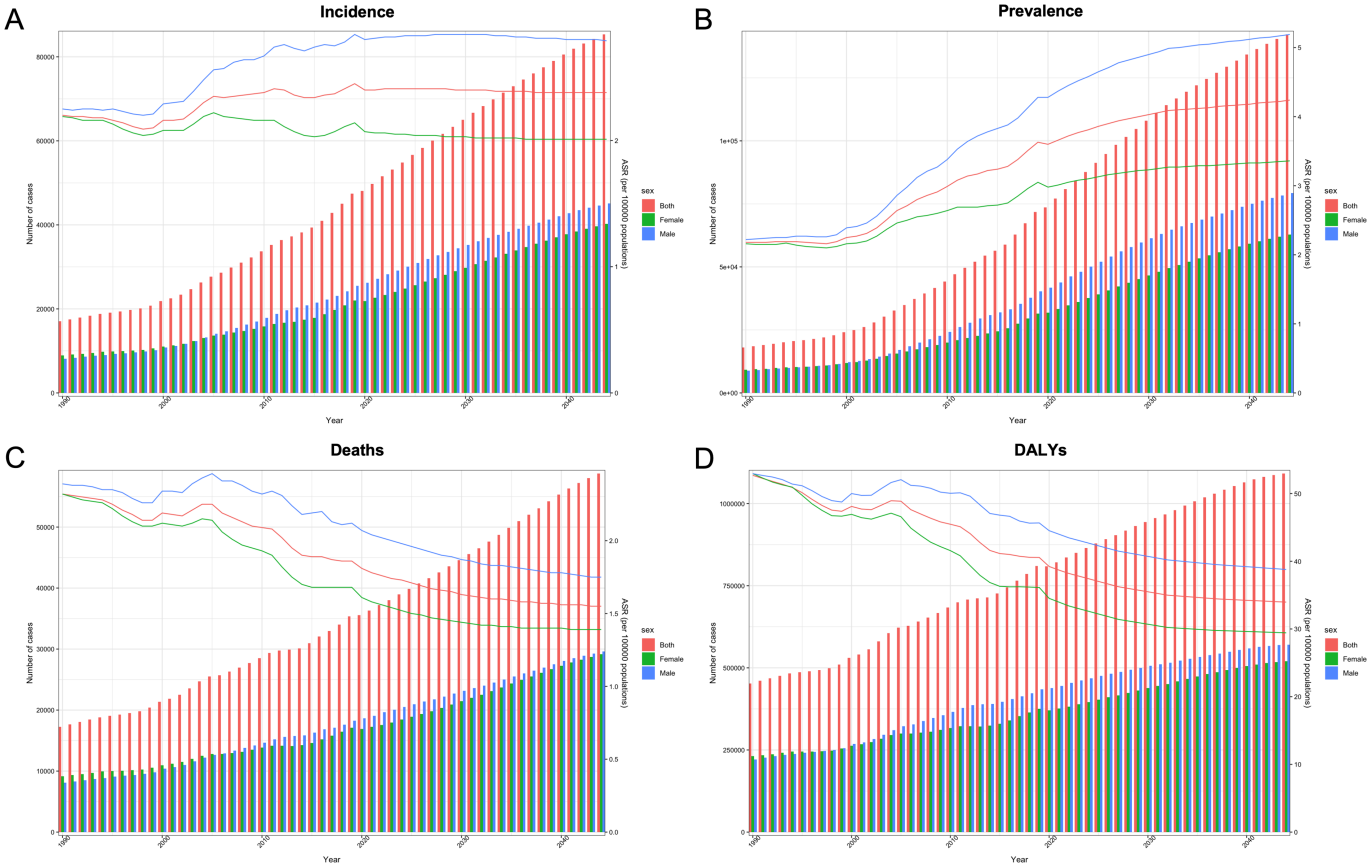
Trends and predictions of the GBTCs disease burden in China from 1990 to 2044 by sex. The lines represent trends, and the columns represent numbers. **(A)** The ASIR and number of incident cases of GBTCs. **(B)** The ASPR and number of prevalent cases of GBTCs. **(C)** The ASMR and numbers of deaths associated with GBTCs. **(D)** The ASDR and number of DALYs associated with GBTCs. ASIR, age-standardized incidence rate; ASPR, age-standardized prevalence rate; ASMR, age-standardized mortality rate; DALYs, disability-adjusted life years; ASDR, age-standardized DALYs rate; GBTCs, gallbladder and biliary tract cancers.

Using the NAPC model, we predicted the ASIRs of sex-specific GBTCs from 2022 to 2044. Our findings indicate a gradual decline in the ASIR for females during this period, whereas males are expected to experience an initial increase followed by a subsequent decrease, albeit with a smaller range of amplitude changes (Figure 4A). Furthermore, we anticipate a substantial increase in the ASPR for both males and females during this period, with a particularly pronounced increasing trend observed among males (Figure 4B). Notably, our predictions indicate a gradual and consistent decline in both ASMR rates and age-standardized DALYs rates attributed to GBTCs across all populations from 2022 to 2044 (Figures 4C,D).

## Discussion

On the basis of data from the GBD 2021, in the present study, we present a comprehensive assessment of the current status in 2021, trends over the past 40 years, and predictions from 2022 to 2044 regarding the burden due to GBTCs in China. As evidenced by our findings, the number of incident cases, prevalent cases, deaths, and DALYs associated with GBTCs in China significantly increased by 3.03-fold, 4.43-fold, 2.19-fold, and 1.90-fold, respectively, from 1990 to 2021. Similarly, the ASIR and ASPR clearly exhibited a consistent upward trend over the 40-year period, whereas the ASMR and ASDR demonstrated a declining pattern. The forecast results from 2022 to 2024 suggest a potential upward trend in the GBTCs-related ASPR while indicating a downward trend in both the ASMR and ASDR. Furthermore, the overall change trend of the ASIR remains relatively stable at a high level. Although the disease burden of GBTCs varies significantly in terms of geographic region, country level, ethnicity, and culture, our findings align with those of previous studies indicating a progressively severe disease burden in East Asia and South Asia (7, 9, 17).

Traditionally regarded as a rare tumor, GBTCs exhibit a remarkably high incidence in China, ranking second among hepatobiliary tumors following hepatocellular carcinoma (1, 17). GBTCs account for approximately 15% of all hepatobiliary tumors in China (18), although significant advancements in surgical techniques have resulted in cholecystectomy, hepatectomy and liver transplantation as curative treatments for GBTCs, several surgical limitations still exist, including tumor stage and primary site (19, 20). Moreover, the potential adverse effects of surgery, such as ischemia–reperfusion injury and infection, significantly impact patient prognosis (21–23). In the context of an increasingly aging population, GBTCs have emerged as a significant challenge to individuals’ well-being. Notably, the ASIR significantly increased for both males and females starting in 1998, with a pivotal turning point observed in 2005. Subsequently, the ASIR continued to demonstrate an upward trend among males but an overall downward trajectory among females. Furthermore, while both sexes exhibited a gradual increase in the ASPR, this trend was notably more pronounced among males. The disparities arise from biological factors (such as male-predominant visceral fat accumulation versus female subcutaneous fat distribution and estrogen protection), behavioral risks (including higher rates of smoking and alcohol use among males), and hormonal influences (such as the loss of estrogen’s protective effects after menopause). These results highlight the necessity for gender-specific prevention strategies that target visceral fat reduction in men and promote metabolic health in postmenopausal women to reduce the risk of GBTCs (24, 25). Despite metabolic, hormonal, and physical differences between males and females, the precise influence of sex on incidence and prevalence rates remains unclear due to significant regional disparities. For example, in East Asia and South Asia, there was a greater disease burden of GBTCs among males than among females, whereas the opposite trend was observed in Latin America (8, 9, 26). The findings of our study also indicate a significantly elevated risk of both incidence and mortality from GBTCs among individuals aged 65 years and above, particularly within the 80–94-year-old age group. The increased susceptibility of the elderly population to gallstones, due to factors such as reduced metabolic capacity and compromised immunity, increases their vulnerability to the development and fatality of GBTCs (10, 27). Moreover, the rapid socioeconomic development in China has not only extended the life expectancy of its population but also given rise to the social issue of population aging, thereby exacerbating the disease burden of GBTCs. Therefore, it is imperative to prioritize disease prevention, early screening, and comprehensive care for the elderly population.

High BMI is now widely acknowledged as a significant risk factor for gastrointestinal tumors, including GBTCs (28). With improvements in living conditions, however, certain lifestyle challenges, such as reduced physical activity and the consumption of high-calorie diets, have emerged. These factors have contributed to an overall increase in obesity rates within society (29, 30). In 2021, China has transitioned from a country with low SDI and World Bank Low Income in 1990 to a country with high-middle SDI and World Bank Upper Middle Income. However, our study revealed a significant increase in the proportion of deaths and DALYs associated with high BMI in 2021 compared with 1990 in China, highlighting the escalating public health challenge posed by obesity and its consequences. Additionally, this phenomenon may be linked to a country’s level of socioeconomic status. Although it has been suggested that the disease burden of GBTCs increases with SDI (9, 26), current observations indicate a rapid increase in the disease burden of GBTCs within regions characterized by a lower SDI. Furthermore, gallstones are strongly related to GBTCs, and high BMI is an independent risk factor for gallstone development (31, 32). Studies indicate a significant familial correlation between gallstones and biliary tract diseases. A family history of gallstones doubles the risk of GBTCs, while individuals with both gallstones and a positive family history face a 57-fold increased risk of GBTCs, suggesting a potential synergistic effect. These findings highlight the critical roles of genetic predisposition and high BMI in the pathogenesis of GBTCs (33). On the one hand, the socioeconomic development of lower-SDI regions has facilitated advancements in medical care, leading to enhanced efficiency in GBTCs screening and diagnosis; on the other hand, the lifestyle and dietary habits of lower-SDI regions have gradually assimilated the high-burden dietary patterns prevalent in higher-SDI regions (34, 35).

The disease burden of GBTCs in China is anticipated to escalate over the next two decades, necessitating the implementation of cost-effective strategies to address this substantial burden. The first step toward enhancing national awareness involves bolstering the dissemination of cancer prevention measures. To mitigate societal and individual burdens, proactive measures should be taken to promote healthy lifestyle behaviors, such as reducing BMI-related risk factors like smoking, unhealthy diets, obesity, and physical inactivity. Targeted interventions tailored for high-risk populations should include weight management programs, early screening initiatives, and lifestyle modifications. In light of the societal pressures stemming from an aging population, future research should prioritize advancing our understanding of GBTCs pathogenesis, with a particular emphasis on genetic and molecular biomarkers. Furthermore, it will be imperative to enhance prognosis by improving treatment technologies and diversifying therapeutic strategies.

The present study is subject to certain limitations. First, we exclusively utilized data from the GBD 2021, which may introduce some bias in terms of data accuracy and stability. Second, the current limited data analysis does not allow for further refinement of the analysis on the basis of subtypes derived from GBTCs. Additionally, in the present study, we considered only a limited number of risk factors, necessitating a broader exploration of potential risk factors associated with GBTCs. Finally, the prediction model should be regarded as a reference tool rather than an exact representation of future trends. Moving forward, we will further investigate the disease burden of GBTCs in clinical cohorts to provide more valuable insights for the formulation of public health prevention policies.

## Conclusion

Our study provides a comprehensive chronological analysis of the disease burden trends of GBTCs in China over the past four decades as well as projections for the next two decades. Additionally, this study highlights sex and age-related disparities and underscores the importance of high BMI as a prominent risk factor for GBTCs. The results of this study provide robust data support for reducing the disease burden of GBTCs in the Chinese population, as well as formulating effective strategies and measures for their prevention and management.

## Data Availability

The raw data supporting the conclusions of this article will be made available by the authors, without undue reservation.

## Footnotes

^1^ https://vizhub.healthdata.org/gbd-results/
